# Do It Well or Not at All: Alternative Flight Solutions for Alpine Insects

**DOI:** 10.1002/ece3.70673

**Published:** 2024-12-03

**Authors:** Graham A. McCulloch, Brodie J. Foster, Travis Ingram, Jonathan M. Waters

**Affiliations:** ^1^ Department of Zoology University of Otago Dunedin New Zealand

**Keywords:** adaptation, elevation, Plecoptera, stoneflies, wing loss

## Abstract

Exposed and isolated alpine ecosystems present evolutionary challenges for flying species worldwide. Many insects have undergone dramatic wing reduction in response to these harsh conditions, losing the ability to fly. By contrast, some taxa have countered alpine conditions by evolving larger wings to improve flight ability. In this study, we investigated how two independent clades of *Zelandoperla fenestrata* stoneflies respond to upland environments. Our results revealed strikingly different adaptations to elevation across the two closely related clades. In Clade 1 (southern South Island), wing length *decreases* sharply with increasing elevation. In contrast, wing length in the geographically adjacent Clade 2 (northern South Island, and North Island) *increases* with elevation. These contrasting strategies highlight the diverse adaptive pathways that may exist even for closely related lineages encountering similar environmental challenges.

## Graphical abstract

Alpine ecosystems present many challenges for flighted insects. Some insects have adapted to these conditions by undergoing dramatic wing reduction while others have evolved larger wings. Here we document both these contrasting adaptations to alpine life within a single insect species, illustrating how evolutionary outcomes are not always predictable based on phylogeny alone.

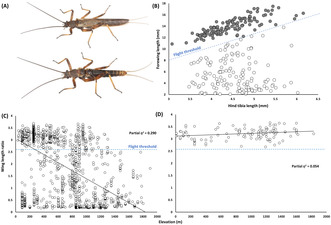

## 1 Introduction

The astonishing diversification of insects has often been attributed to the evolution of flight (Wagner and Liebherr 1992), yet this dispersal capacity has subsequently been lost across numerous insect lineages (Roff 1990). Wing loss is particularly common in alpine ecosystems and other insular habitats (Hodkinson 2005) and has frequently been linked to environmental variables such as physical isolation (Roff 1990), wind exposure (Darwin 1859; McCulloch, Foster, Ingram, et al. 2019; Leihy and Chown 2020), or air temperature and pressure (Roff 1990). Intriguingly, however, alpine lineages of some insect taxa appear to buck this trend (e.g., Odonata; Mayr 1963; Moore and Khan 2023; *Drosophila*: Stalker and Carson 1948), instead showing substantial increases in wing size with increasing elevation. It is thought that larger wings move more air downward with each wingbeat, potentially lowering the metabolic demands of flight in alpine environments (Dillon, Frazier, and Dudley 2006; Dudley 2002).

**FIGURE 1:**
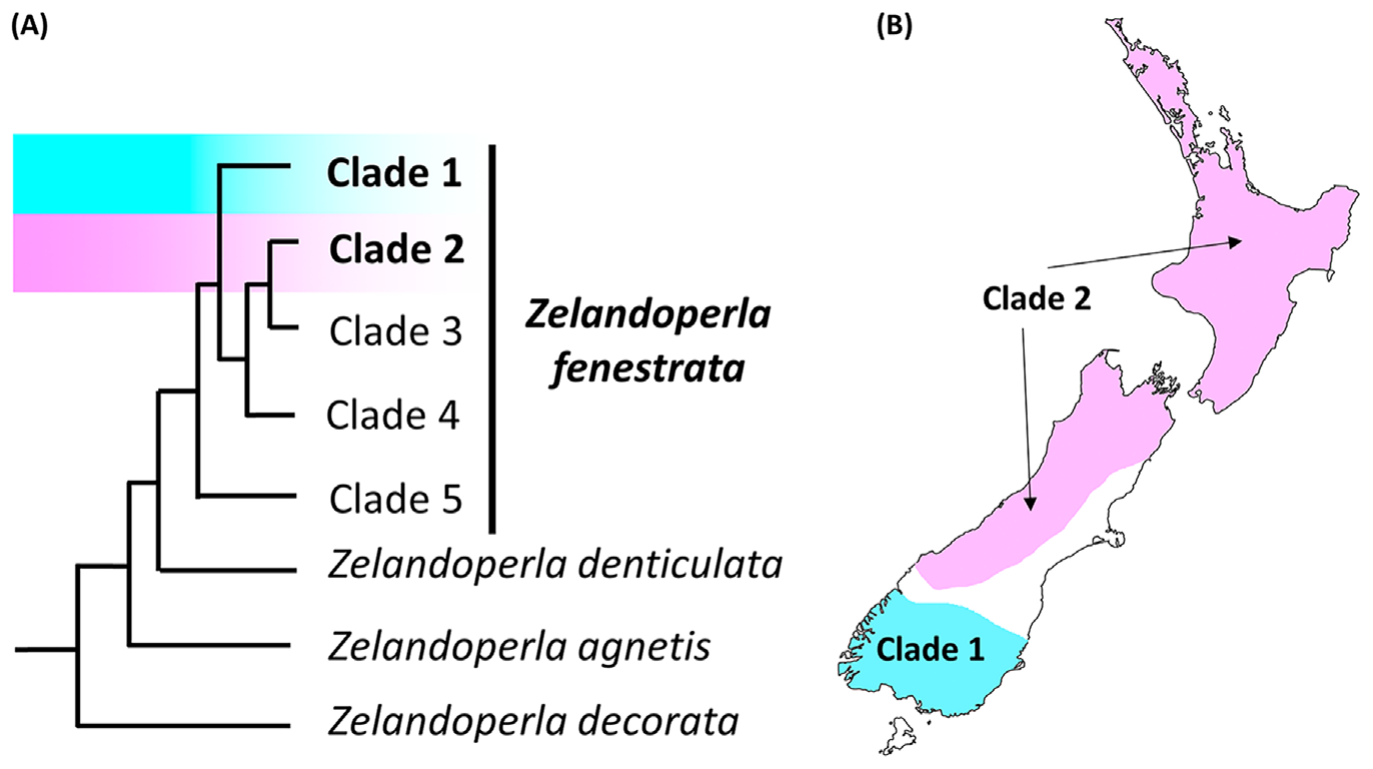
The phylogenetic relationships among the five *Zelandoperla fenestrata* clades (A), and the geographic distributions of Clades 1 and 2 (B; modified from McCulloch, Wallis, and Waters 2009).

Shifts in flight ability are particularly common features of New Zealand's alpine stonefly assemblages (McLellan 1976; McCulloch and Waters 2018; McCulloch, Foster, Ingram, et al. 2019; McCulloch et al. 2021). The widespread stonefly *Zelandoperla fenestrata* Tillyard is wing dimorphic (McLellan 1976; McCulloch, Wallis, and Waters 2009), so presents an ideal model system to examine how insects respond to alpine conditions. This species is distributed in streams throughout New Zealand and is divided into five geographic clades, two of which are widespread (Clades 1 and 2; Figure 1; McCulloch, Wallis, and Waters 2009).

Here we examine the relationship between elevation and wing phenotype in two closely related *Z. fenestrata* clades (Clade 1 and Clade 2) to test for geographic variation in elevational adaptation. Because both clades encounter broadly similar environmental conditions in alpine environments, we hypothesise that these two closely related clades will respond similarly to elevation gradients.

## 2 Materials and Methods

**FIGURE 2:**
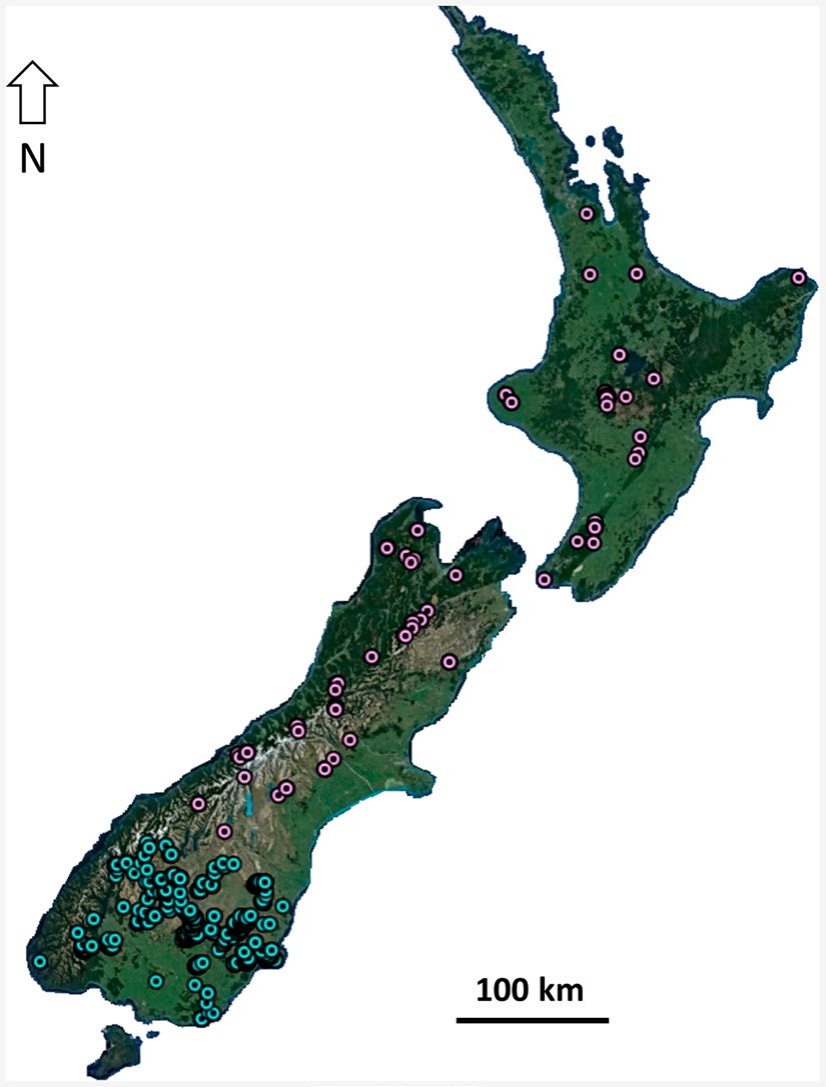
Collection localities for 1858 Clade 1 *Zelandoperla fenestrata* specimens (cyan) and 112 Clade 2 *Z. fenestrata* specimens (pink). Details of each sampling locality are in Appendix S1.

We obtained phenotypic measurements from adult *Z. fenestrata* species complex specimens from a wide range of elevations across the distributions of Clades 1 and 2 (McCulloch, Wallis, and Waters 2009; Figure 2). A total of 1972 preserved specimens (967 females, 1005 males) were examined across 377 unique locations originating from field collections, two private collections and five institutional collections: Auckland War Memorial Museum, New Zealand Arthropod Collection (Auckland), Museum of New Zealand Te Papa Tongarewa (Wellington), Canterbury Museum (Christchurch) and Otago Museum (Dunedin).

We photographed stoneflies using a stereo microscope and measured forewing length and femur length using a stage micrometer in ImageJ (Schneider, Rasband, and Eliceiri 2012). We used these measurements to calculate the forewing: hind tibia length ratio (wing length ratio). This ratio influences flight ability in insects (Dudley 2002), and crucially is directly linked to flight capability in *Z. fenestrata* , with stoneflies unable to fly if their wing length ratio is below 2.57 (Foster et al. 2021).

We fit a linear model to test whether elevation, clade assignment or their interaction influenced wing length ratio. We included sex and island as additional predictors; they were not of specific interest but were treated as fixed effects as they have only two levels each. We tested hypotheses using the ‘Anova’ function in the ‘car’ package (Fox and Weisberg 2019) in R (R Core Team 2024). Given that there was an interactive effect of elevation and clade assignment that complicated the interpretation of the main effects, we split the data by clade assignment. Within each clade, we tested for effects of elevation on wing length ratio, including sex and (for Clade 2) Island as additional predictors. We calculated partial *η* ^2^ as a measure of effect size using the ‘etasq’ function in the ‘heplots’ (Friendly 2007).

## 3 Results and Discussion

**FIGURE 3:**
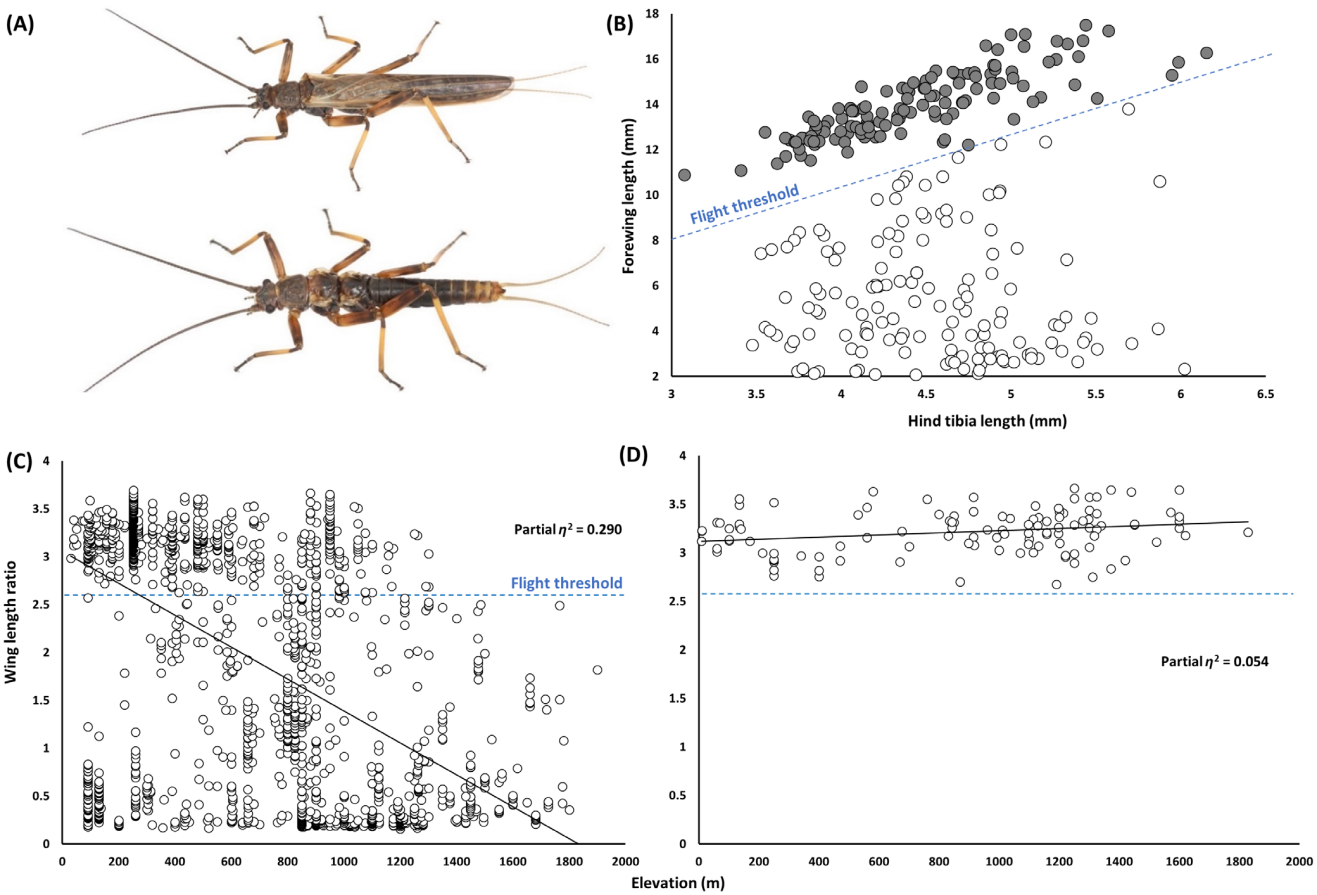
Alternative flight solutions for alpine stoneflies. (A) Full‐winged and wing‐reduced *Zelandoperla fenestrata* ecotypes. (B) The experimentally determined flightless threshold in *Z. fenestrata* (modified from Foster et al. 2021). Gray = flighted, white = flightless (C) A negative association between elevation and wing length ratio (forewing length: hind tibia length) in Clade 1 *Z. fenestrata* (D) A positive association between wing length ratio and elevation in Clade 2 *Z. fenestrata* .

Our phenotypic analyses across the range of the *Z. fenestrata* complex reveal regional contrasts in relationships between wing phenotype and elevation, manifesting as a weak interactive effect of elevation and clade assignment on wing length ratio (*F*_1,1965_ = 68.1, *p* < 0.00001, partial *η* ^2^ = 0.030). Specifically, as elevation increases, wing length in Clade 1 (southern South Island) *decreases* rapidly below the experimentally determined flightless threshold (*F*_1,1854_ = 771.7; *p* < 0.00001; partial *η* ^2^ = 0.290; Figure 3B,C; see Foster et al. 2021). These results support the findings of several previous studies testing for elevational clines in stonefly wing length (McCulloch, Foster, Dutoit, et al. 2019; Foster et al. 2021; Rendoll‐Carcamo et al. 2023), and follow the expectations of Darwin (1859) (i.e., enhancing local recruitment in isolated windy ecosystems; see McCulloch, Foster, Ingram, et al. 2019).

In contrast, wing length in the geographically adjacent Clade 2 (northern South Island, and North Island) weakly *increases* with elevation (*F*_1,110_ = 6.33; *p* = 0.013; partial *η* ^2^ = 0.054; Figure 3D), following the predictions of Mayr (1963) (i.e., overcoming the metabolic and aerodynamic challenges of alpine flight). These contrasting elevational clines thus appear to represent contrasting adaptive solutions for two closely related lineages faced with near‐identical ecological gradients. The weak strength of association between wing length and elevation in Clade 2 *Z. fenestrata* suggests that factors other than elevation may contribute to the relatively minor phenotypic variation present in this clade (in which all individuals appear to be flight capable (Figure 3)).

The contrasting elevational clines among clades could potentially reflect corresponding contrasts in abiotic features, with the southern region (Clade 1) more often subject to harsh alpine conditions (e.g., low temperatures, snow cover, wind) than the more northern region occupied by Clade 2. Alternatively, it remains possible that Clade 2 has simply yet to evolve the genetic capacity for wing reduction that is clearly widespread in the widely polymorphic Clade 1. Future studies should assess zones of contact between these lineages to test for the possibility of localised adaptive introgression (Brauer et al. 2023) of flight‐reduction alleles between clades. Further research should also examine whether alpine representatives of Clade 2 exhibit any additional morphological adaptations to enhance flight in alpine environments, such as alterations in wing shape (see Montejo‐Kovacevich et al. 2019; Hernández‐L et al. 2010) or wing loading (i.e., body weight to wing area ratio; see Lozier et al. 2021).

Recent genomic analyses have suggested that ‘predictable’ evolutionary responses to similar selective gradients (e.g., Blount, Lenski, and Losos 2018) may be heavily shaped by standing genetic variation (i.e., repeated sorting of ancestral alleles in different populations; Waters and McCulloch 2021). In the case of *Z. fenestrata* , widespread flight loss events in Clade 1 are likely explained at least in part by repeated selection on standing variation (McCulloch et al. 2021). However, the current study reveals completely contrasting evolutionary responses to similar elevational gradients across distinct clades of *Z. fenestrata* ; one involving flight loss (see Hodkinson 2005), and the other apparent flight enhancement (see Moore and Khan 2023). These findings thus imply that evolutionary outcomes may be phylogenetically less predictable than previously suggested (Blount, Lenski, and Losos 2018), with even closely related lineages adapting in different ways to comparable selective regimes (Losos 2011; Gould 2002).

## Author Contributions

**Graham A. McCulloch:** conceptualization (equal), formal analysis (equal), funding acquisition (lead), investigation (lead), visualization (lead), writing – original draft (supporting), writing – review and editing (equal). **Brodie J. Foster:** conceptualization (equal), data curation (lead), formal analysis (equal), investigation (equal), writing – review and editing (supporting). **Travis Ingram:** formal analysis (equal), writing – review and editing (equal). **Jonathan M. Waters:** conceptualization (supporting), funding acquisition (supporting), investigation (supporting), supervision (lead), writing – original draft (lead), writing – review and editing (equal).

## Conflicts of Interest

The authors declare no conflicts of interest.

## Supporting information


Appendix S1.

## Data Availability

Raw data associated with the research are available in Appendix S1.
